# Cancer Genes Mutations in Benign Metastasizing Leiomyoma: A Case Report

**DOI:** 10.7759/cureus.5154

**Published:** 2019-07-16

**Authors:** Thu-Cuc Nguyen, Peter A Drew, Long H Dang, Cai Yuan

**Affiliations:** 1 Internal Medicine, University of Central Florida, Orlando, USA; 2 Pathology, University of Florida, Gainesville, USA; 3 Hematology and Oncology, Ochsner Health System, Baton Rouge, USA; 4 Hematology and Oncology, University of Florida, Gainesville, USA

**Keywords:** benign metastasizing leiomyoma, cancer genes mutations

## Abstract

Benign metastasizing leiomyoma is a very uncommon clinicopathologic entity with unknown molecular pathogenesis. We present a case of a 40-year-old woman who has a history of surgical resection of a large uterine leiomyoma and then subsequently presented with benign metastasizing leiomyomas to her lungs. Due to her tumor being estrogen receptor (ER) positive and progesterone receptor (PR) positive, she was empirically treated with anastrozole with sustained clinical benefit. Molecular studies with Foundation One testing showed low mutational burden and mutational variants in five known cancer genes. Our findings have important clinical and pathogenetic implication for metastasizing uterine leiomyoma.

## Introduction

Benign metastasizing leiomyoma (BML) is characterized by indolent clinical presentation with metastatic leiomyoma nodules [1]. Most presentations involve the lungs, but may also involve lymph nodes, heart, brain, skin, and eye [2-4]. For the majority of the time, patients presented without symptoms and have incidental findings of small pulmonary nodules on chest X-ray or computed tomography scan (CT scan), but also sometimes presented with coughs, shortness of breath, hemoptysis, pleural effusions, or pneumothorax [5-7]. The pathogenesis of BML has not been clearly defined. The natural history of presentation of BML fits either possibilities, that BML may be metastasis from uterine leiomyoma cells or in situ hormone-sensitive proliferation of smooth muscle cells [8]. Many women have a history of prior hysterectomy for uterine leiomyomas, which is consistent with the first hypothesis and others may not have prior gynecological surgery, which is consistent with the second hypothesis [9]. On pathology, the tumor appears benign, has low mitosis index, does not have evidence of anaplasia or necrosis, and has minimal vascularization [10]. BML typically expresses estrogen and progesterone receptors, favoring their potential uterine origin [11]. Potential treatments for BML include close surveillance, surgical resection of metastatic nodules, and systemic hormonal approaches targeting estrogen receptor/progesterone receptor (ER/PR) including hysterectomy with bilateral salpingo-oophorectomy, ER/PR modulators, aromatase inhibitors (AI), and gonadotropin-releasing hormone agonists [12-13]. To date, there have not been any publications regarding molecular pathways or mutational analyses in BML to suggest novel therapeutic approaches.

## Case presentation

**Figure 1:**
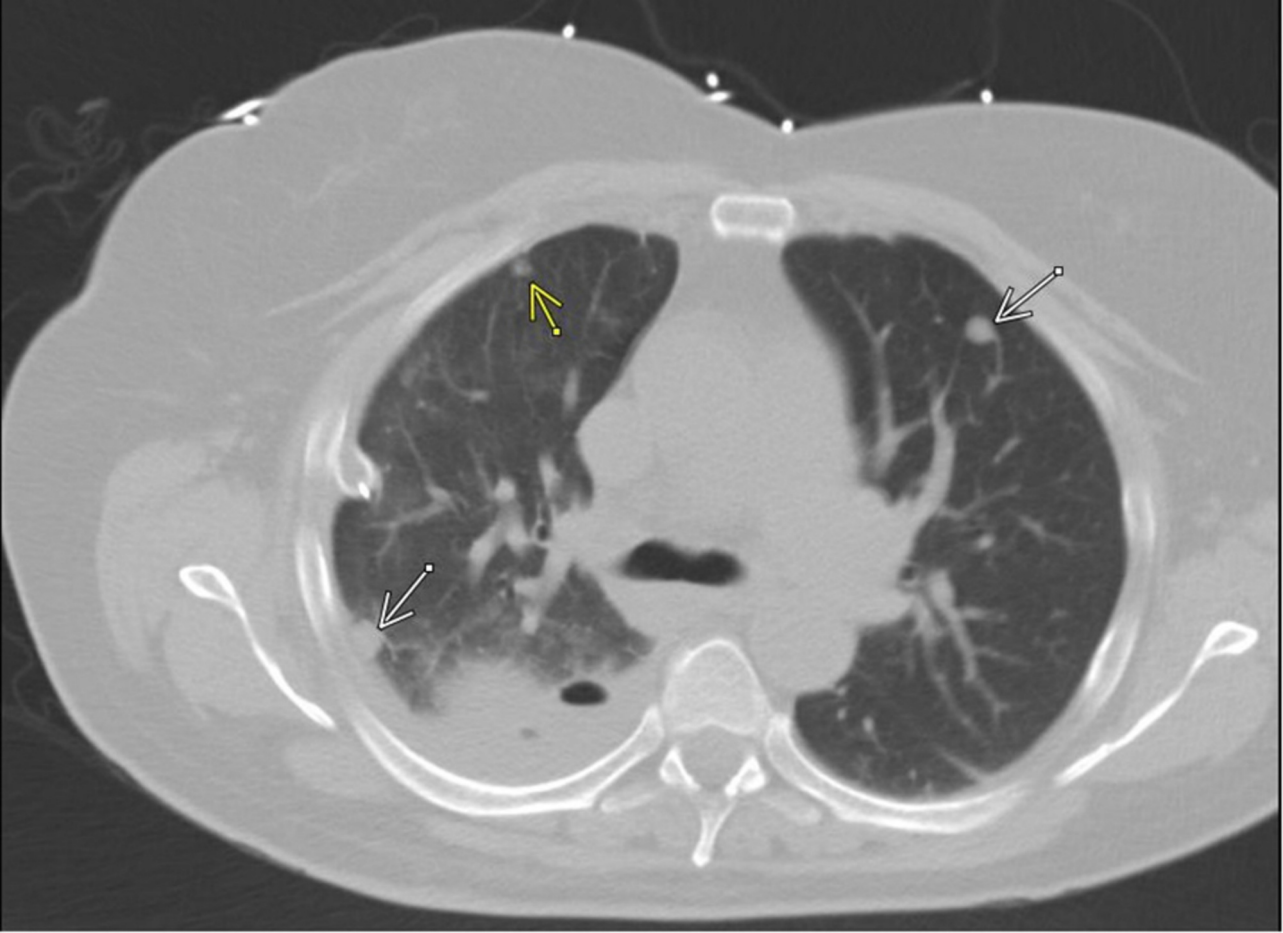
Chest CT scan 1/2015: Large right loculated pleural effusion, with multiple bilateral pulmonary nodules.

A 59-year-old female presented on November 10, 2014, with shortness of breath. She did not have significant past medical history other than a total hysterectomy due to uterine fibroids many years ago. On presentation, a chest CT scan showed a large right pleural effusion. She had thoracentesis and 1.2 liters of pleural fluid removed. Bronchoscopy showed atelectasis of right lower lung lobe. All bronchial washings and biopsies were negative for cancer cells. Cultures were also negative. About one month later, repeat chest CT scan showed a large loculated right pleural effusion, with multiple bilateral pulmonary nodules concerning for metastatic disease (Figure 1).

Repeat thoracentesis was unsuccessful due to loculation. She subsequently underwent right middle lobe wedge resection.

**Figure 2:**
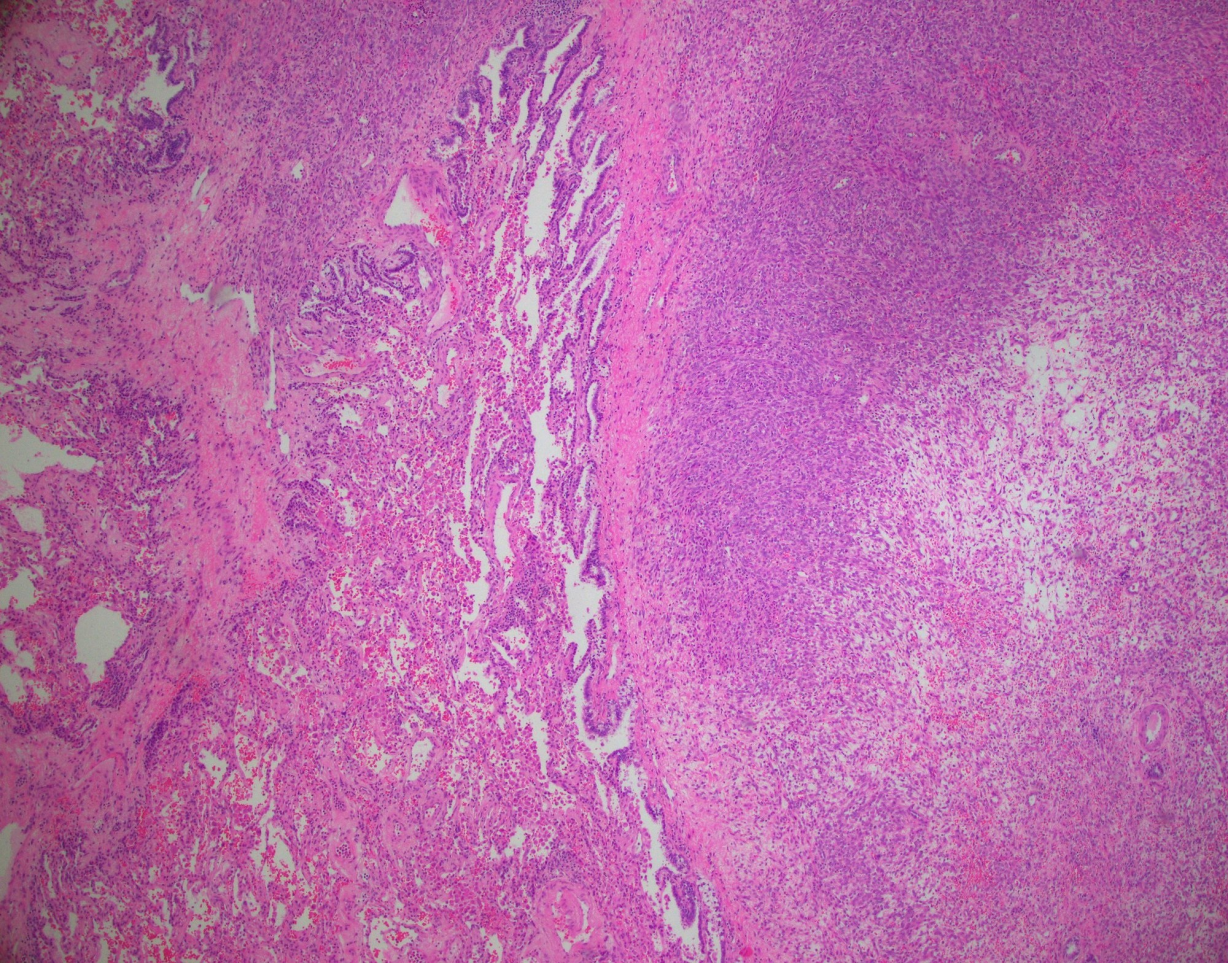
The H&E slide at 4x magnification shows the metastatic spindle cell tumor at the right and the non-neoplastic lung tissue on the left. H&E: Hematoxylin and eosin

**Figure 3:**
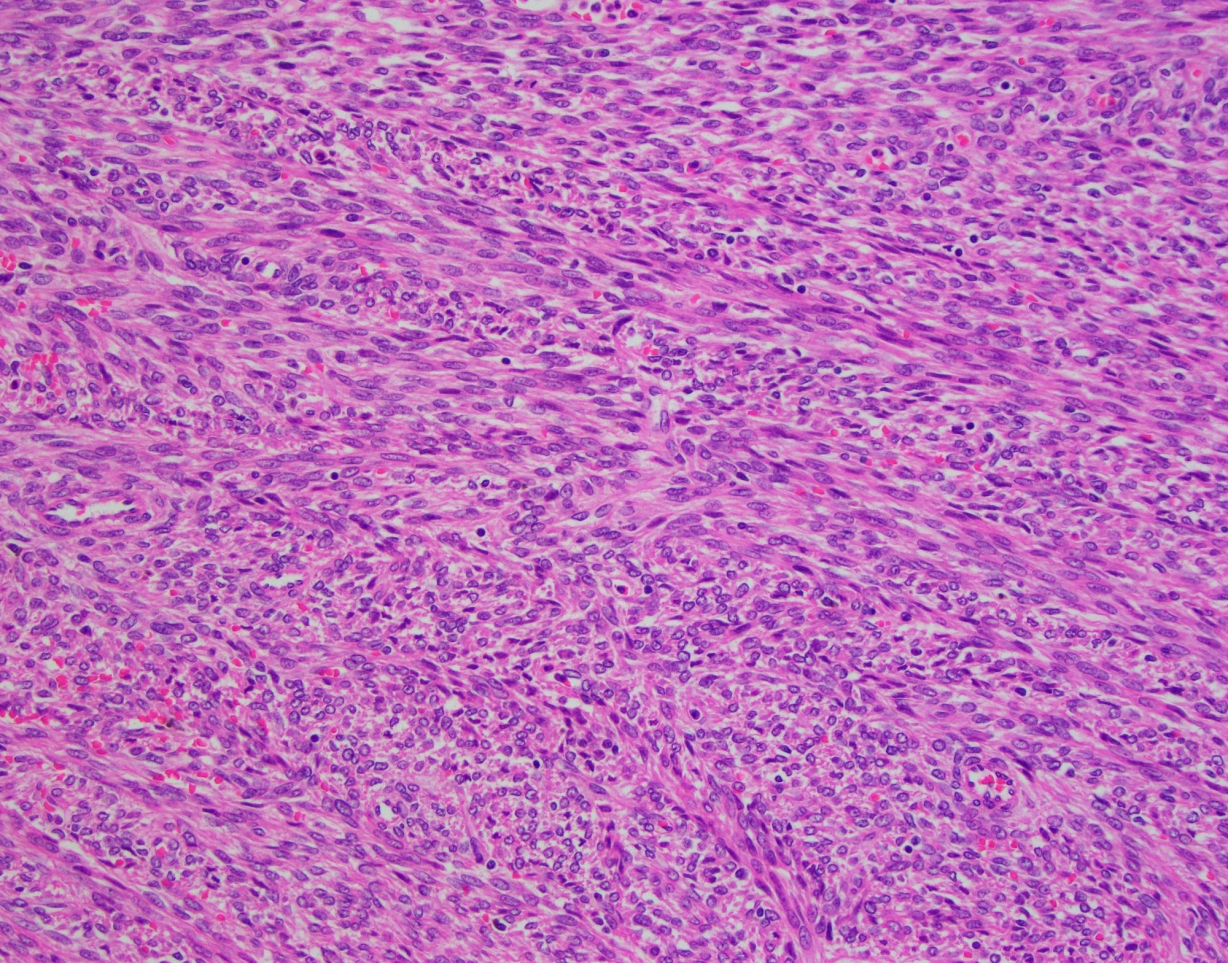
The H&E slide at 20x magnification shows the spindle cell tumor at higher power. The spindle cells are arranged in a fascicular growth pattern. The nuclei are "cigar" shaped. Mitoses were present, but the mitotic index was less than 10 mitoses per 10 high power fields. No tumor necrosis or significant cytologic atypia were noted. H&E: Hematoxylin and eosin

Histological examination of the lesion consists of well-demarcated cellular spindle cell arranged in a fascicular growth pattern. No tumor necrosis or significant cytologic atypia were noted. Mitoses were present, but the mitotic index was less than ten mitoses per ten high power fields (Figures 2, 3).

**Figure 4:**
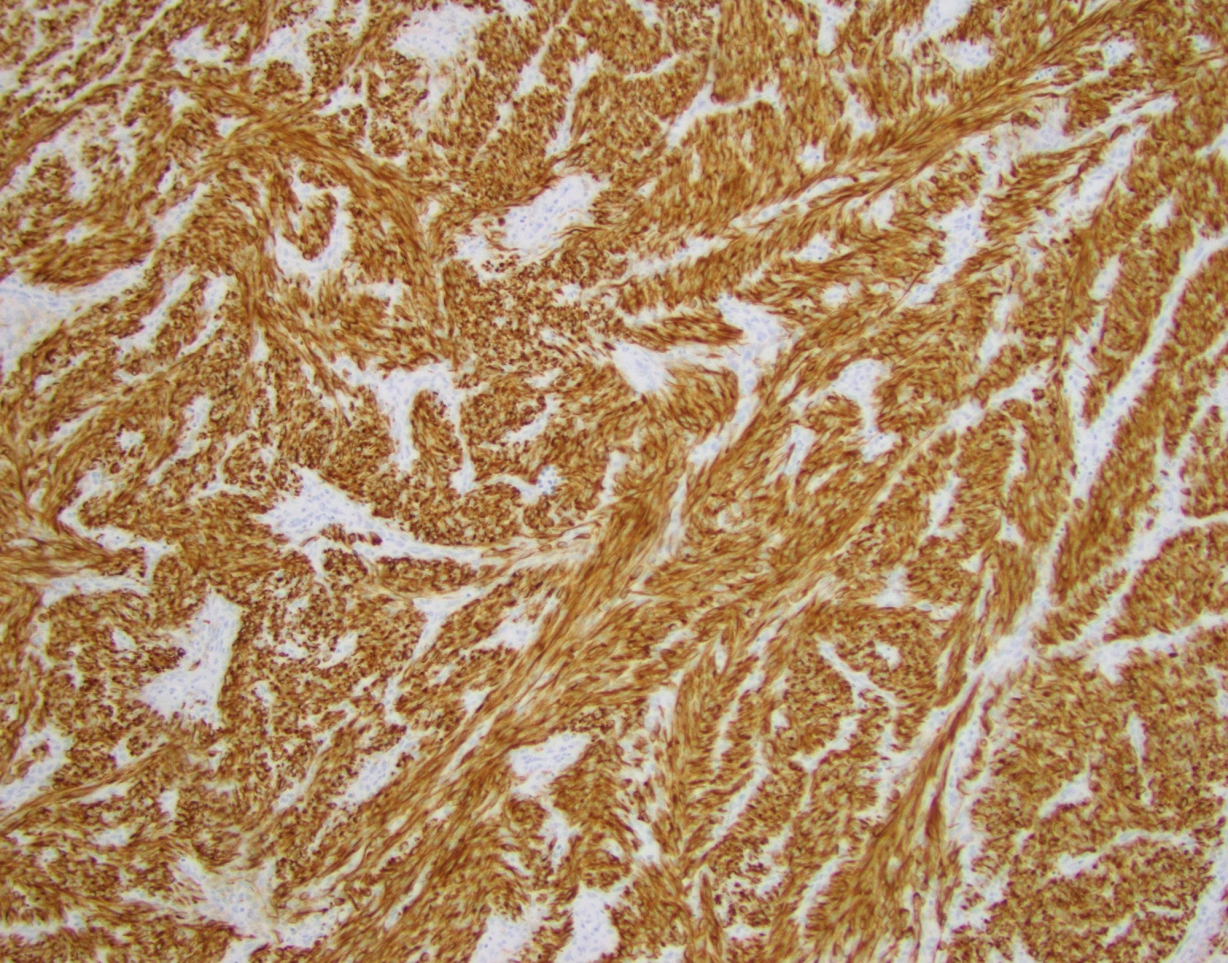
Immunostains show the tumor is positive for desmin.

**Figure 5:**
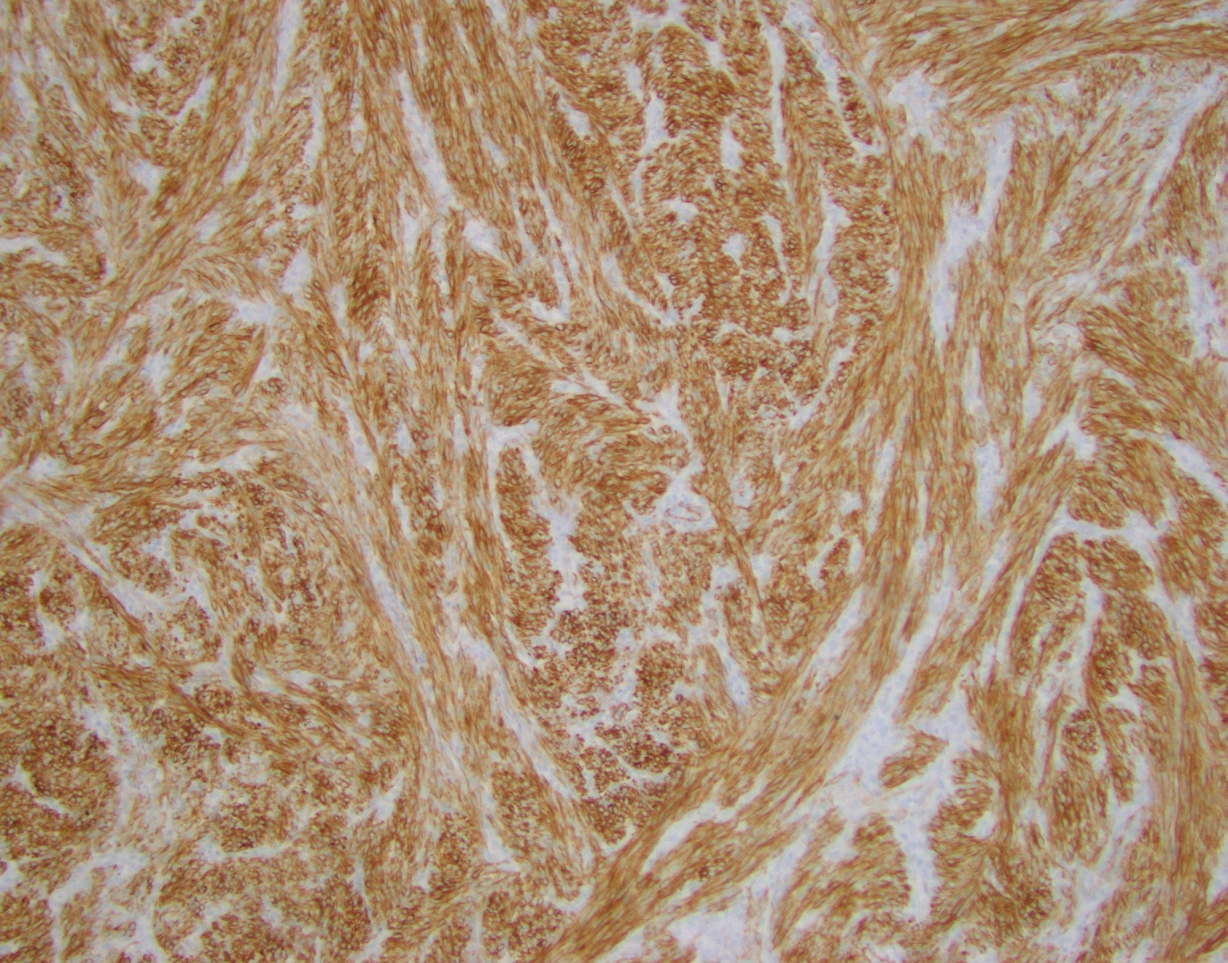
Immunostains show the tumor is positive for muscle actin.

**Figure 6:**
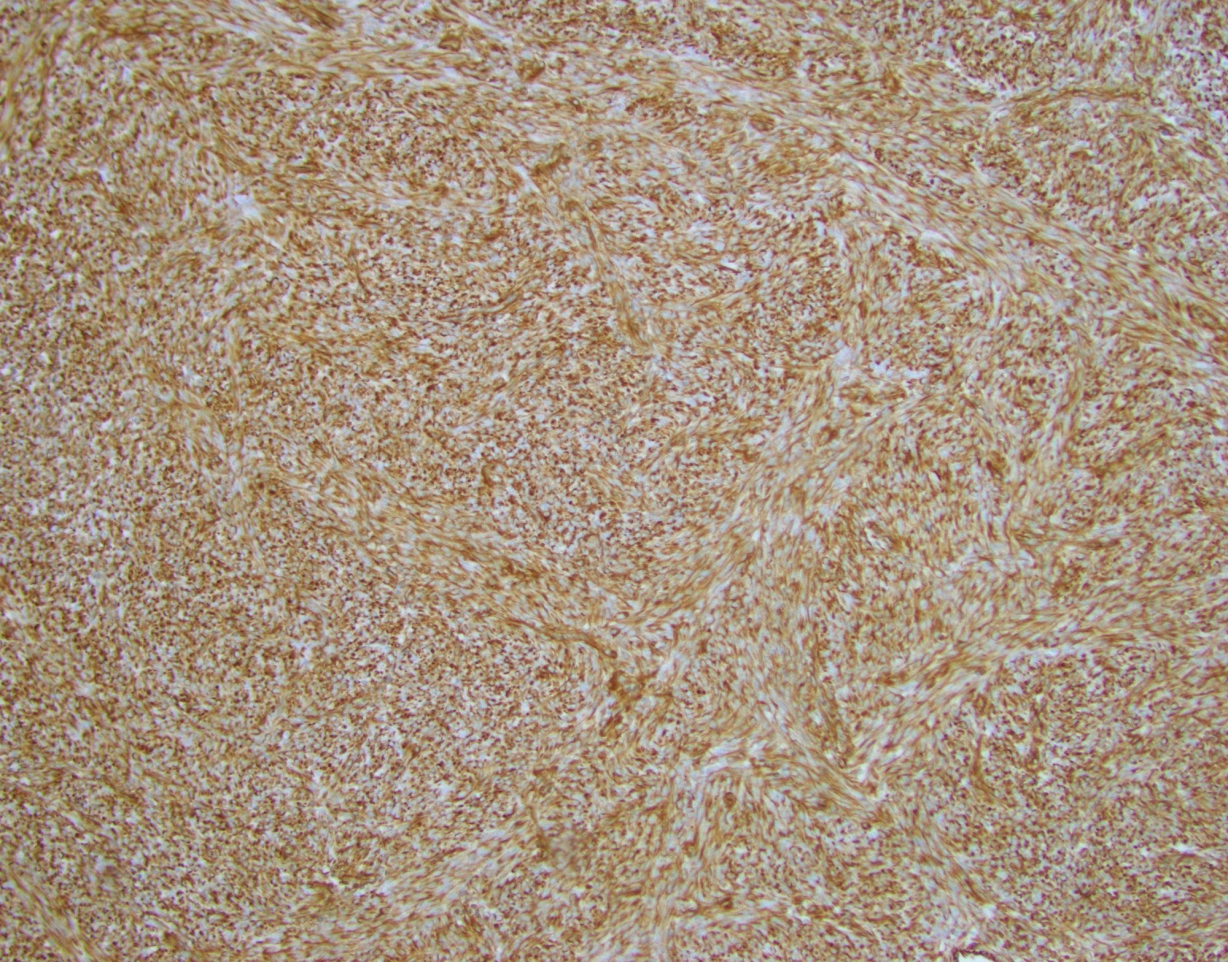
Immunostains show the tumor is positive for vimentin.

Immunostains showed the tumor is positive for desmin (Figure 4), muscle actin (Figure 5), vimentin (Figure 6), and ER+/PR+, and negative for S-100, pan-cytokeratin, CD 31 and CD34, consistent with a benign metastasizing leiomyoma.

**Figure 7:**
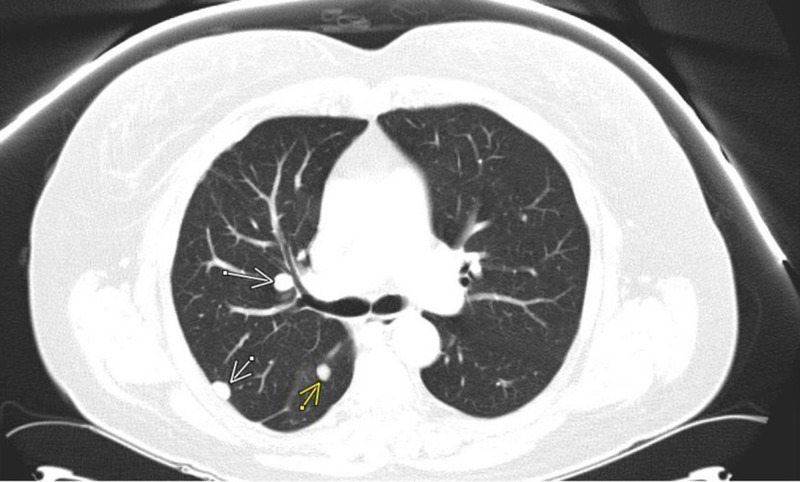
Chest CT scan 10/2015 showed pulmonary nodules increased in size.

After surgery, the patient underwent regular surveillance. Ten months later, pulmonary nodules were noted to increase in size on surveillance CT scan (Figure 7).

**Figure 8:**
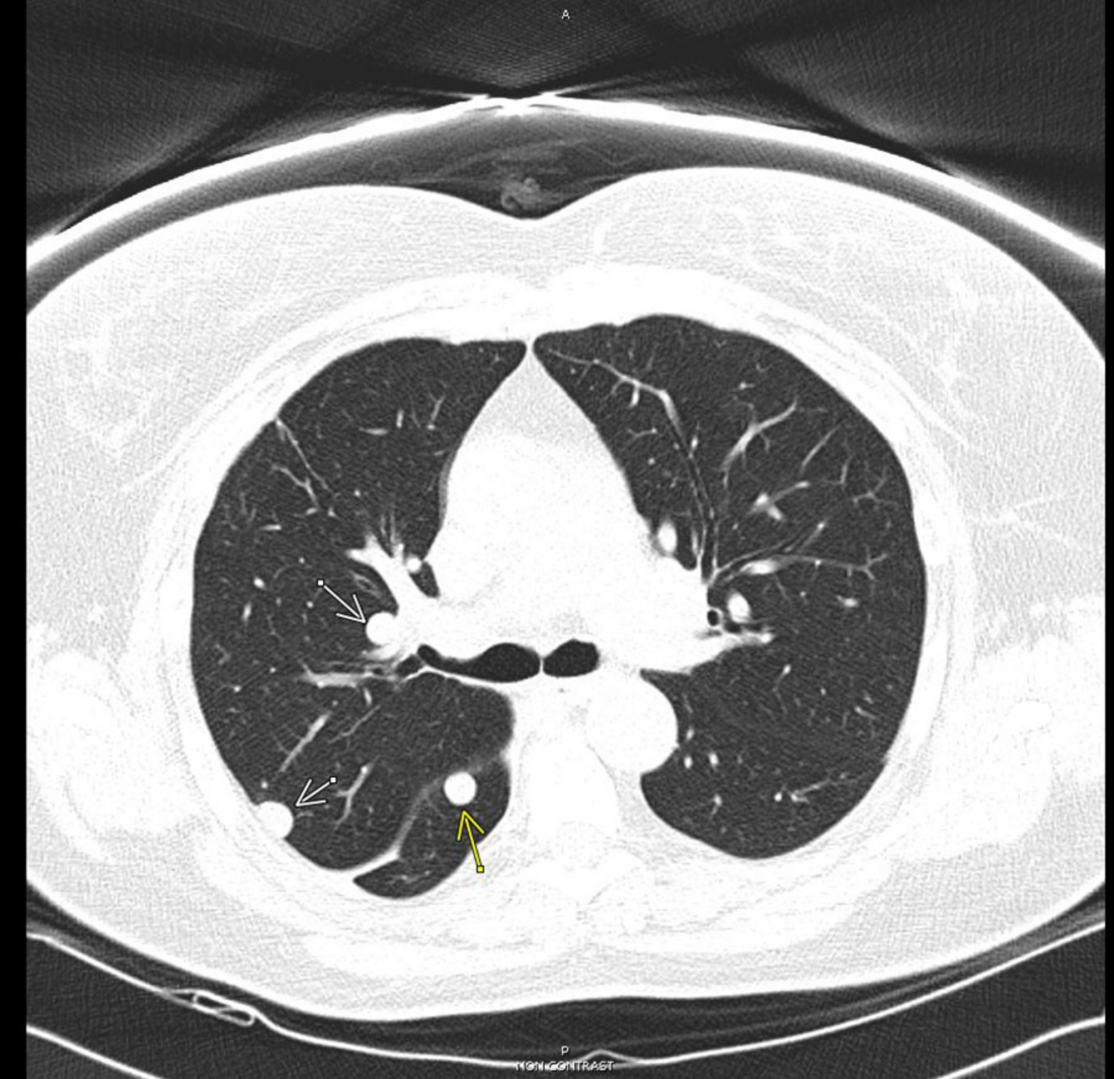
10/2016 chest CT scan showed pulmonary nodules stable in size.

The patient was started on anastrozole 1 mg once daily. Her pulmonary nodules have been stable for the past four years and there is no recurrence of pleural effusion (Figure 8).

To investigate for possible targetable mutations for future therapy, her tumor was sent for Foundation One testing. DNA sequencing was done on 406 genes along with selected introns from 31 genes involved in rearrangement, and RNA sequencing was evaluated on 265 genes. There was low tumor mutation burden (TMB) with mutations noted in five genes: ARID2 (N1340K), BCL11B (amplification), NTRK1 (R6W), TCL1A (amplification), and XPO1 (rearrangement).

## Discussion

Clinical course of BML is heterogenous, from typically very indolent asymptomatic disease to aggressive disease with rapid progression, pulmonary compromise, and death. Lung is the most common involved site, but in addition other sites have been described including mediastinum, nervous system, skin, and bone. On radiology evaluation, patients can have solitary, multiple nodular densities in both lungs, or even miliary pattern; and nodules can be solid, cavitary, or cystic. Prognosis depends on rate of progression and responsiveness to treatment. Time interval between hysterectomy and BML presentation is typically 15 years [1-7]. In an evaluation of 57 patients reported in the literature, survival from time of BML diagnosis is by average 11.6 years [14].

Current treatment for BML depends on clinical presentation whether symptomatic or asymptomatic, disease burden, and disease course. For low burden, slow growing, asymptomatic disease, close surveillance without treatment would be the most common option. On the other hand, for high burden, fast growing, symptomatic disease, treatment would be needed. Due to dependence on estrogen for tumor progression, treatments centered on inhibiting estrogen biosynthesis or the estrogen receptors on BML. Estrogen receptor inhibition includes tamoxifen, and estrogen biosynthesis pathway inhibition includes surgical oophorectomy, medical oophorectomy with GnRH agonists or progestins, or peripheral estrogen synthesis inhibition with aromatase inhibitors [15].

To better understand the pathogenesis of BML and develop new treatment options, there has been a lot of interests in studying the molecular pathways for BML. Various methodologies have been used on archival BML samples including cytogenetics analysis, single nucleotide polymorphism array analysis, and whole exome sequencing analysis. Chromosomal level changes have been identified including 19q and 22q terminal deletions, loss of 1p and 13q material, and 6p21 rearrangement [16]. Point mutations have been identified in the BMP8B gene and MED12 genes [17,18]. We have identified five novel mutations in our BML patient: ARID2 (N1340K), BCL11B (amplification), NTRK1 (R6W), TCL1A (amplification), and XPO1 (rearrangement).

Cancer progression and development of resistance to therapy are due to accumulation of genetic changes or new mutations [19]. Mutational analysis with Foundation One testing is a commercially available method to query for mutations in known cancer genes. Our mutational analysis of the patient’s tumor prior to exposure to anastrozole may indicate cancer genes and pathways that are important in transformation, and may be targetable for treatment. Unfortunately, the analysis did not reveal any actionable mutation for a targeted drug. TMB is also low, suggesting that immune therapy with PD1/PDL1 inhibitors or CTLA4 inhibitor would not be a good approach.

## Conclusions

The patient is currently in complete remission on treatment with anastrozole, an aromatase inhibitor. Anastrozole is well-tolerated and can be continued based on efficacy without dose-limiting toxicities. Upon progression on anastrozole, it would be informative to biopsy a progressing lesion to see if there are new mutations in cancer genes or pathways that are responsible for tumor progression and resistance to anastrozole, and perhaps targetable by currently available inhibitors. Future mutational testing in BML should expand to include more genes.
